# Pathogenic Role of Fibrinogen in the Neuropathology of Multiple Sclerosis: A Tale of Sorrows and Fears

**DOI:** 10.1007/s11064-023-03981-1

**Published:** 2023-07-13

**Authors:** Mubarak Alruwaili, Hayder M. Al-kuraishy, Athanasios Alexiou, Marios Papadakis, Barakat M. ALRashdi, Omnya Elhussieny, Hebatallah M. Saad, Gaber El-Saber Batiha

**Affiliations:** 1 Department of Internal Medicine, College of Medicine, Jouf University, Sakaka, Saudi Arabia; 2 Department of Pharmacology, Toxicology and Medicine, Medical Faculty, College of Medicine, Al-Mustansiriyah University, P.O. Box 14132, Baghdad, Iraq; 3 Department of Science and Engineering, Novel Global Community Educational Foundation, Hebersham, NSW 2770 Australia; 4 AFNP Med, 1030 Vienna, Austria; 5 Department of Surgery II, University Hospital Witten-Herdecke, University of Witten-Herdecke, Heusnerstrasse 40, 42283 Wuppertal, Germany; 6 Biology Department, College of Science, Jouf University, Sakaka, 41412 Saudi Arabia; 7 Department of Histology and Cytology, Faculty of Veterinary Medicine, Matrouh University, Marsa Matruh, 51744 Egypt; 8 Department of Pathology, Faculty of Veterinary Medicine, Matrouh University, Marsa Matruh, 51744 Egypt; 9 Department of Pharmacology and Therapeutics, Faculty of Veterinary Medicine, Damanhour University, Damanhour, 22511 Egypt

**Keywords:** Multiple sclerosis, Demyelination, Oxidative stress, Metformin

## Abstract

Multiple sclerosis (MS) is an autoimmune demyelinating neurodegenerative disease of the central nervous system (CNS) due to injury of the myelin sheath by immune cells. The clotting factor fibrinogen is involved in the pathogenesis of MS by triggering microglia and the progress of neuroinflammation. Fibrinogen level is correlated with MS severity; consequently, inhibition of the fibrinogen cascade may reduce MS neuropathology. Thus, this review aimed to clarify the potential role of fibrinogen in the pathogenesis of MS and how targeting of fibrinogen affects MS neuropathology. Accumulation of fibrinogen in the CNS may occur independently or due to disruption of blood–brain barrier (BBB) integrity in MS. Fibrinogen acts as transduction and increases microglia activation which induces the progression of inflammation, oxidative stress, and neuronal injury. Besides, brain fibrinogen impairs the remyelination process by inhibiting the differentiation of oligodendrocyte precursor cells. These findings proposed that fibrinogen is associated with MS neuropathology through interruption of BBB integrity, induction of neuroinflammation, and demyelination with inhibition of the remyelination process by suppressing oligodendrocytes. Therefore, targeting of fibrinogen and/or CD11b/CD18 receptors by metformin and statins might decrease MS neuropathology. In conclusion, inhibiting the expression of CD11b/CD18 receptors by metformin and statins may decrease the pro-inflammatory effect of fibrinogen on microglia which is involved in the progression of MS.

## Introduction

Most of neurodegenerative diseases including Alzheimer’s disease (AD) and Parkinson’s disease (PD) are age-related disorders that are most common in elderly subjects [1]. However, multiple sclerosis (MS) is the most common demyelinating neurodegenerative disease of the central nervous system (CNS) in young adults [2]. Of note, MS was initially described by Jean-Martin Charcot a French neurologist in 1868 who illustrated multiple scars in the brain and spinal cord [3]. It has been shown that MS interrupts motor and sensory neuronal signal transmission leading to motor and sensory deficits [4]. MS is characterized by specific symptoms including vision loss in one eye, double vision, muscle weakness, and motor-sensory incoordination [5]. Notably, MS may be progressive or relapsing forms in which the symptoms disappear and return [5]. Of interest, MS affects about 2.8 million people globally with the difference among many populations; MS is more common in women at 20–50 years [6]. Remarkably, MS is not a curable disease, and 85% of MS cases presented as an isolated clinical syndrome, 45% have motor or sensory dysfunctions, 20% of MS patients have optic neuritis, and 10% presented with brainstem disorders [7]. Particularly, 85% of MS patients presented with acute exacerbations followed by improvement [8]. Nevertheless, 15% of MS patients presented with gradual motor-sensory dysfunction without a period of recovery [8]. However, a combination of these two forms may occur, and relapse is triggered by several factors including viral infections and stress [7, 8]. Symptoms of MS are increased during exposure to high temperatures [9]. An acute attack of MS is treated by corticosteroids and/or plasmapheresis [10]. However, chronic MS is managed by disease-modifying treatments like interferons, glatiramer, and mitoxantrone [11].

**Fig. 1:**
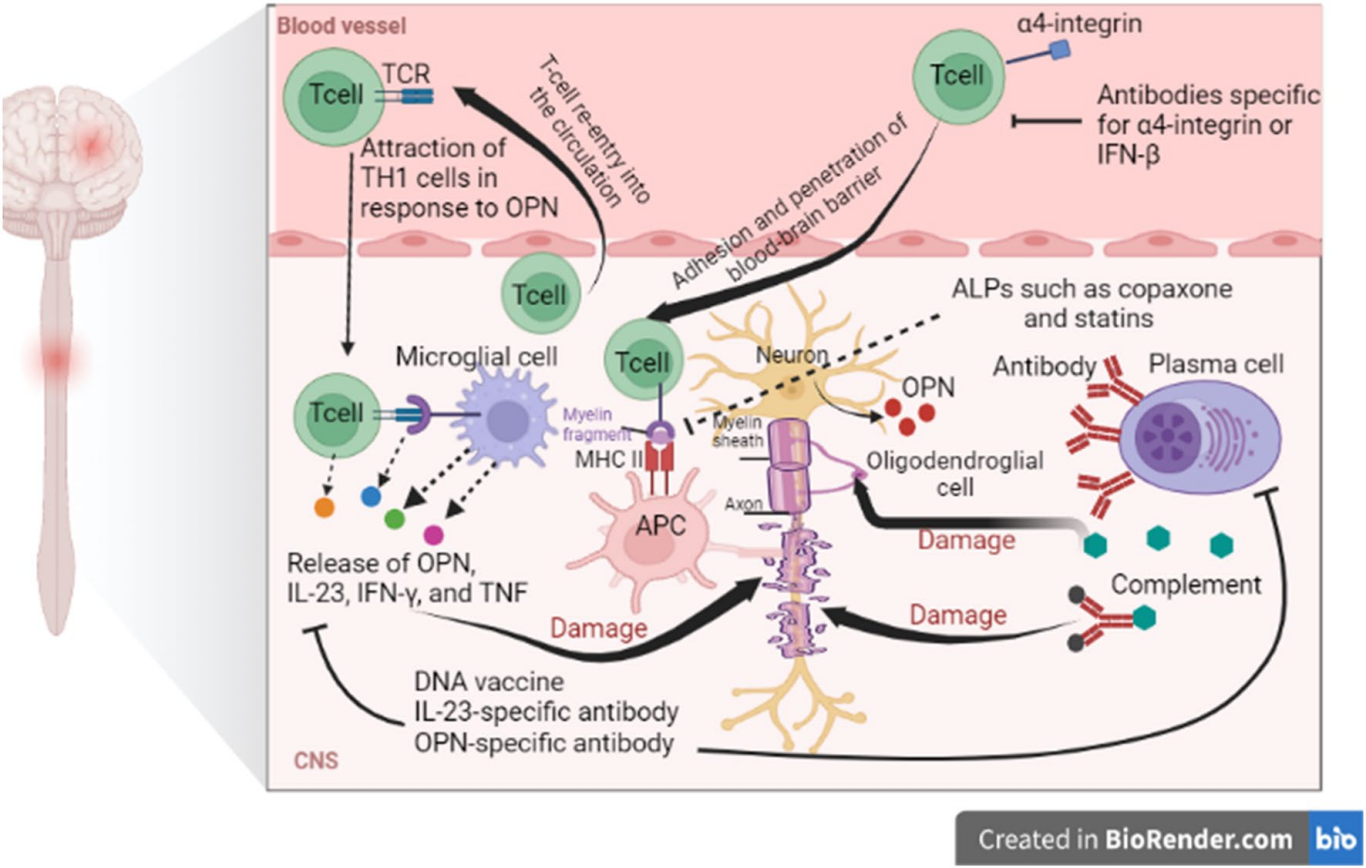
Pathophysiology of multiple sclerosis (MS): entry of autoreactive T cells which interact with microglia and active release of pro-inflammatory cytokines and osteopontin. Besides, autoreactive T cells antigen-presenting cells (APCs) expressing major histocompatibility II (MHCII) lead to injury of oligodendrocytes and disruption of neuronal myelin sheath with the development of multiple sclerosis (MS)

Furthermore, MS is regarded as an autoimmune disease causing the injury of myelin sheath by immune cells and inhibiting the production of myelin [12]. Oligodendrocytes which are concerned with the synthesis of the myelin sheath are mostly affected in MS [13, 14]. Progressive loss of myelin sheath with axonal injury leads to neuronal dysfunction [15]. Partial remyelination in the remission state and demyelination in the relapse phase lead to sclerotic lesions in the CNS [16]. Besides, reactive astrocytosis in response to neuronal injury promotes plaque formation [8]. Moreover, MS plaques are numerous focal areas of demyelination scattered in the brain’s white matter and spinal cords as well as in the deep grey matter and cerebral cortex [17]. In the MS plaques, there is a large infiltration of immune cells including T lymphocytes, monocytes, B cells, and plasma cells [18]. It has been shown that inflammation acts as a central role in the pathogenesis of MS due to the uninhibited activation of T lymphocytes [19]. Peripheral auto-reactive T lymphocytes prompt inflammatory changes in the MS [20]. However, the underlying mechanism for the activation of peripheral auto-reactive T lymphocytes is not fully elucidated. Polyclonal activation of peripheral auto-reactive T lymphocytes by viral antigens or molecular mimicry may be the convincing mechanism [21]. Peripheral auto-reactive T lymphocytes can cross the blood–brain barrier (BBB) by binding integrin on the immune cells and vascular cell adhesion protein 1 (VCAM-1) on the endothelial cells [21]. Expression of integrin and VCAM-1 are increased by inflammation and pro-inflammatory cytokines [22, 23]. T-cell-induces the expression and release of matrix metalloproteinase (MMPs) which augment the entry of T cell which is also involved in the degeneration of myelin components [24]. After the entry of peripherally auto-reactive T lymphocytes, they bind to MHCII expressed by dendritic and antigen-presenting cells leading to the reactivation of T cells toward pro-inflammatory phenotype [25]. These changes provoke disruption of myelin components and the release of other CNS antigens with subsequent recruitment of other immune cells and production of specific myelin auto-antibodies which support further injury and loss of myelin sheath [26]. These immune-inflammatory reactions cause further injury of BBB that promotes entry of auto-reactive T lymphocytes and generation of soluble factors which attack synaptic regions causing neuronal dysfunction [27, 28] (Fig. 1).

**Fig. 2:**
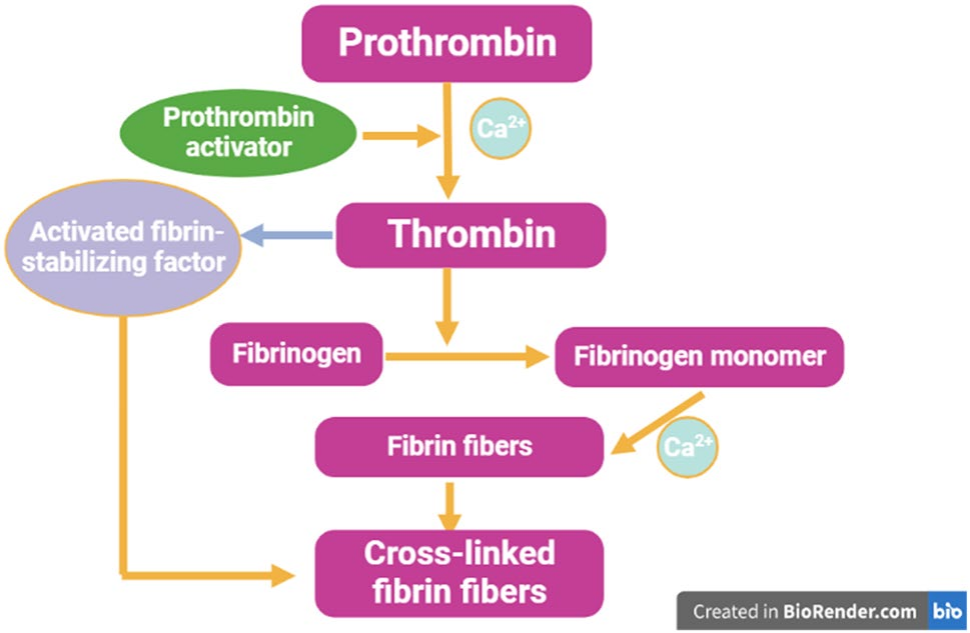
Fibrinogen pathway: prothrombin is converted with the assistance of calcium by prothrombin activator to thrombin which enhances the conversion of fibrinogen to fibrinogen monomer. The formed fibers are cross-linked with the assistance of an activated fibrin stabilizing factor

It has been reported that fibrinogen is involved in the pathogenesis of MS by activating microglia and the development of neuroinflammation [29]. Fibrinogen also called factor I is a 340 kDa glycoprotein produced by hepatocytes and circulates in the blood [30]. The main function of fibrinogen is controlling of blood homeostasis during vascular injury and tissue damage [30]. Fibrinogen was initially discovered by Paul Morawitz in 1905. The normal circulating fibrinogen level is 3 g/L which increases to 4.5 g/L during pregnancy [31]. Fibrinogen is converted by thrombin to fibrin and the formation of blood clots during vascular injury to prevent bleeding [31, 32] (Fig. 2).

Acquired and congenital hypofibrinogenemia is associated with bleeding tendency; however, hyperfibrinogenemia is linked with thrombotic disorders [33]. Fibrinogen is regarded as an acute-phase protein in response to systemic inflammation in different inflammatory disorders [34]. Hyperfibrinogenemia in response to inflammation and malignancy induces thromboembolic disorders [35]. In addition, fibrinogen forms a bridge between platelets via binding fibrinogen receptors (GpIIb/IIIa) on platelets [36]. Acquired dysfibrinogenemia is developed due to dysfunction of fibrinogen that developed in chronic disorders including cirrhosis and chronic hepatitis [37]. Diseased liver produced dysfunctional fibrinogen due to a defect in the glycosylation process of amino acids [38]. Furthermore, circulating autoantibodies in autoimmune disorders interferes with fibrinogen causing the development of dysfunctional fibrinogen [39]. As well, some medications like glucocorticoids and isotretinoin interfere with fibrinogen function [40]. Mutation of fibrinogen induces the production of abnormal fibrinogen involved in the development of familial renal amyloidosis [40]. However, fibrinogen in familial renal amyloidosis is not associated with bleeding or thrombosis [40]. It has been revealed that fibrinogen level is correlated with MS severity [41]. Therefore, inhibition of the fibrinogen cascade may reduce MS neuropathology. Thus, this review aimed to clarify the potential role of fibrinogen in the pathogenesis of MS and how targeting of fibrinogen affects MS neuropathology.

## Fibrinogen and MS

Fibrinogen has pleiotropic effects and plays a critical role in the pathogenesis of MS by induction of inflammatory process and neuroinflammation [11]. Deposition of fibrinogen in the CNS precedes neuroinflammation in MS [41]. However, accumulation of fibrinogen in the CNS may occur due to disruption of BBB integrity in MS [29, 41]. Disturbance of BBB integrity in MS may precede the development of brain lesions that may promote the entrance of plasma proteins including fibrinogen [29, 41]. It has been demonstrated that extracellular vesicles from blood plasma can induce experimental autoimmune encephalomyelitis in mice due to the activation of CD8 T cells by fibrinogen [42]. Likewise, analysis of extracellular vesicles from blood plasma from MS patients showed a higher concentration of fibrinogen [42]. Therefore, fibrinogen is implicated in the development and progression of MS through the induction of neuroinflammation and disease relapse. Fibrinogen is not merely the indicator of BBB injury in MS but acts as transduction increases microglia activation via triggering expression of αvβ3 integrin on CD11b/CD18 [43]. Likewise, brain fibrinogen induces the progression of inflammation, oxidative stress, and neuronal injury [44]. Interestingly, brain fibrinogen impairs the remyelination process by inhibiting the differentiation of oligodendrocyte precursor cells [45]. In vivo study using photon microscopy demonstrated that the clustering of microglia in the perivascular space induced by fibrinogen is developed before the progression of demyelination in MS [46]. Ghorbani and Yong [47] observed that the extracellular matrix acts as a possible modifier of remyelination and neuroinflammation. Furthermore, fibrinogen and fibrinogen-like 1,2 are associated with neuroinflammation and MS neuropathology [48]. Furthermore, fibrinogen can act as an immunomodulatory in the CNS, triggering neuroinflammation and demyelination in MS [49]. A case-control study included 119 MS patients and 68 healthy controls observed that single gene polymorphism 455G/A and VH1299R variant in the fibrinogen gene was associated with higher MS risk [49]. Davalos et al. [50] revealed that deposition of fibrinogen in the CNS triggers the pathogenesis of MS. Many postmortem studies revealed that deposition of fibrinogen in the brain perivascular regions was observed not only in the active sclerotic lesions but also in the pre-active brain lesions mainly in the white and gray matter of cerebral cortex. It has been observed that fibrinogen plasma level was increased in MS patients [40]. An observational cohort study involving 58 MS patients showed that fibrinogen plasma level was increased in patients with active MS and positively correlated with CNS lesions [40]. This study indicated that fibrinogen plasma level is a sensitive biomarker for the early detection of MS.

Of interest, the deposition of fibrinogen in the perivascular space activates microglia and releases pro-inflammatory cytokines causing BBB dysfunction and the progression of MS [40]. A cohort study illustrated that CSF fibrinogen was low in chronic MS patients as compared to other inflammatory disorders involving the CNS [51]. However, in acute MS with injury of BBB integrity, plasma fibrinogen level is increased [51]. Furthermore, increased fibrinogen level in the proteome of platelets was observed in patients with progressive MS [52]. This finding indicated that platelet hyper-reactivity is augmented in MS patients due to overexpression of fibrinogen. Notably, platelets play a critical role in the progression of inflammation in MS [53]. Platelet hyper-reactivity is implicated in the progression of autoimmunity and inflammation in the early phase of MS neuropathology [53]. Therefore, the pro-thrombotic state in MS increases the risk of stroke and cardiovascular complications [54]. Besides, fibrinogen interacts with other coagulation factors in the pathogenesis of MS [54]. A case-control study observed that MS patients were associated with platelet hyperactivity compared to controls [55]. Platelet hyperactivity in MS could be due to endothelial injury and the release of endothelial microparticles which reflect activated T cells in the endothelium [56]. In MS, there is a profound platelet hyper-responsiveness to the different physiological activators leading to intravascular thrombosis [57]. Platelet hyper-responsiveness in MS is also due to hyperinflammation and oxidative stress. Pro-oxidant and pro-inflammatory state in MS also promotes platelet aggregation [57]. It has been shown that activated platelets are highly abundant in MS lesions [53]. Markedly, platelets play a role in the activation of the coagulation cascade through the induction of local generation of thrombin and release of stored clotting factors [58]. In addition, platelets can synthesize fibrinogen, von-Willebrand factor, thrombosthenin, thrombospondin, and membrane glycoproteins [59]. Of note, fibrinogen binds platelets GPIIb–IIIa receptors leading to platelet aggregation and thrombosis [60]. Therefore, activated platelets in MS are highly susceptible to the effect of fibrinogen leading to platelet aggregation.

**Fig. 3:**
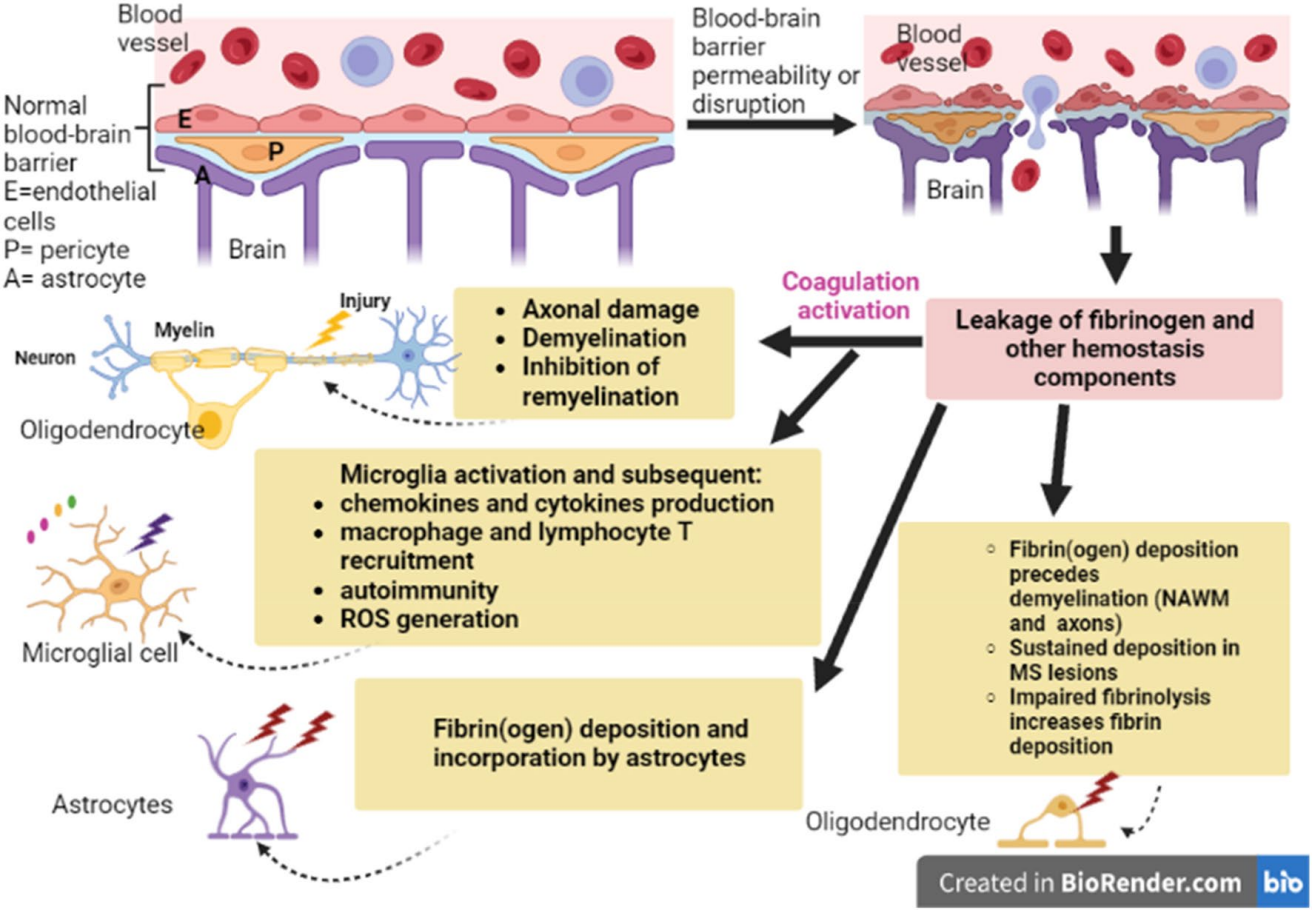
Role of fibrinogen in MS: disruption of the blood–brain barrier (BBB) enhances leakage of fibrinogen from blood vessels. Deposited fibrinogen in the multiple sclerotic lesions leads to the activation of microglia and the release of pro-inflammatory cytokines. As well, fibrinogen triggers demyelination and inhibits remyelination

These findings proposed that fibrinogen is associated with MS neuropathology through interruption of BBB integrity, induction of neuroinflammation, and demyelination with inhibition of the remyelination process by suppressing oligodendrocytes (Fig. 3).

### Oxidative Stress and Fibrinogen

**Fig. 4:**
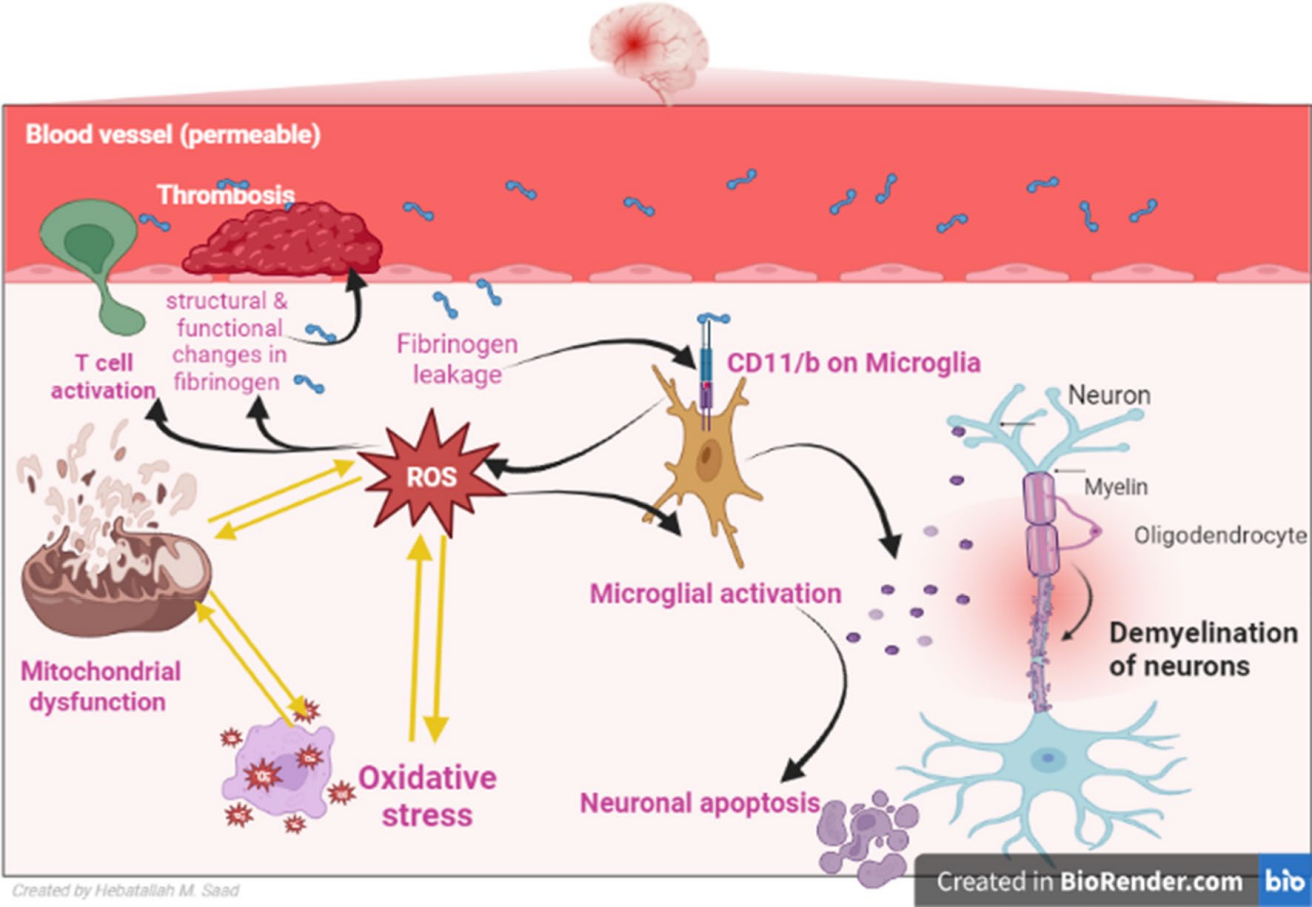
Oxidative stress/mitochondrial dysfunction/fibrinogen in multiple sclerosis (MS): fibrinogen binds the CD11b receptor in microglia leading to the activation of NADPH oxidase and the release of Reactive oxygen species (ROS) leading to mitochondrial dysfunction and injury of oligodendrocytes. Additionally, oxidative stress induces structural and functional changes in fibrinogen leading to thrombosis. ROS trigger peripheral activation of T cells and the development of autoreactive T cells. ROS triggers the activation of microglia and induction of neuronal apoptosis

Oxidative stress plays an essential role in the pathogenesis of MS through the induction of the demyelination process [61]. Reactive oxygen species (ROS) trigger peripheral activation of T cells and the development of autoreactive T cells. ROS triggers the activation of microglia and induction of neuronal apoptosis [61]. Inflammatory reactions in MS can aggravate oxidative stress burst in the activated macrophages and microglia leading to neuronal demyelination. In sequence, oxidative stress augments the propagation of inflammation in MS [62]. Consequently, there is positive feedback activation between oxidative stress and inflammation in MS. A case–control study showed that biomarkers of oxidative stress were increased in patients with relapsing–remitting MS compared to healthy controls [63]. These findings proposed that oxidative stress can aggravate inflammatory reactions and contribute to more neuronal injury and progression of MS (Fig. 4).

On the other hand, oxidative stress induces structural and functional changes in fibrinogen leading to more risk of thrombosis [64]. In addition, oxidative modification of fibrinogen increases its propensity for spontaneous activation and progression of thrombosis [65]. Chronic oxidative stress and inflammation predispose to the development of venous thromboembolism [66]. A cohort study that included patients with suspected venous thromboembolism illustrated that nitrated fibrinogen level was higher in patients who develop venous thromboembolism compared to patients that were not developed venous thromboembolism [66]. This finding suggests that nitrated fibrinogen could be a possible biomarker of venous thromboembolism. However, mitochondrial superoxide enhances oxidative modification and fibrinogen proteolysis leading to coagulopathy during chronic inflammatory conditions [67]. Interestingly, leaky BBB in AD enhances fibrinogen in the brain leading to the activation of inflammation and oxidative stress through the stimulation of microglia [68]. Further, fibrinogen binds the CD11b receptor in microglia leading to the activation of NADPH oxidase and the release of ROS [69]. Therefore, selective deletion of the brain CD11b receptor prevents fibrinogen-induced microglia activation and attenuates ROS-mediated neurotoxicity in mice [70]. These verdicts indicated a potential interaction between oxidative stress and fibrinogen in MS neuropathology. Thus, higher oxidative stress in MS provokes oxidative modification of fibrinogen which in turn triggers oxidative stress and inflammation in MS.

### Mitochondrial Dysfunction and Fibrinogen

It has been reported that mitochondrial dysfunction is involved in neuronal loss in MS due to unrestrained activation of microglia and associated neuronal injury [71]. Of note, the impairment of mitochondrial permeability transition pore by Ca^2+^ dyshomeostasis and ROS is the central mechanism for the progress of mitochondrial dysfunction in MS [72]. In addition, the pathological opening of mitochondrial permeability transition pores in response to nitrogen species, Ca^2+^ and ROS, provoke an influx of many solutes into the mitochondrial matrix causing matrix expansion and mitochondrial rupture, and cell deaths [72]. Furthermore, mitochondrial dysfunction is regarded as a vital trigger of programmed axon death in MS [73]. Uric acid and serum lactate are considered potential biomarkers of mitochondrial dysfunction [74]. A case-control study included 32 MS patients and 20 healthy controls revealed that lactate serum level but not serum uric acid was augmented in MS patients compared to the controls [74]. It has been anticipated that mitochondrial dysfunction modifies lymphocyte homeostasis causing a defective apoptotic process of auto-reactive T cells to allocate them to perpetuate within the CNS and maintain an inflammatory cycle in MS patients [75]. Thus, activation of Th1 cells and their lymphokines like interferon-gamma (INF-γ) and IL-2 which induce the transformation of B-lymphocyte to plasma cells generate autoantibodies against myelin antigens [75]. As a result, mitochondrial dysfunction might be a primary cause for MS progression via alteration of lymphocyte activity, or a secondary result due to oxidative stress caused by MS (Fig. 4).

Notably, mitochondrial dysfunction and the release of mitochondrial DNA induce the release of ROS which causes the oxidation of lipoprotein and plasma proteins like fibrinogen leading to the development of atherosclerosis [76]. An experimental study illustrated that fibrinogen interrupts mitochondrial membrane potential and the development of mitochondrial dysfunction in mice with burn injury [77]. In a cross-sectional study involving patients with sarcopenia which developed due to mitochondrial dysfunction, plasma fibrinogen level and its products were increased compared to healthy controls [78]. This observation suggests that increasing fibrinogen levels is associated with the development and progression of mitochondrial dysfunction. In addition, higher fibrinogen level in preeclampsia is correlated with disease severity due to the induction of mitochondrial dysfunction and inflammation [53]. Of note, chronic inflammation is associated with the development of mitochondrial dysfunction [79]. In this state, fibrinogen acts as a link between immunoinflammatory response and the development of mitochondrial dysfunction. Herein, fibrinogen is linked with the direct development of mitochondrial dysfunction or indirectly through the induction of inflammation.

### Inflammatory Signaling Pathways and Fibrinogen

Diverse types of inflammatory signaling pathways and receptors including nuclear factor kappa B (NF-κB), nod-like receptor pyrin 3 receptor (NLRP3) inflammasome, and toll-like receptor 4 (TLR4) are implicated in the pathogenesis of MS [80].

#### Toll-Like Receptor 4

Toll-like receptor 4 (TLR4) is an innate immune sensor aware of the immune system to the presence of external pathogens [81]. Activation of TLR4 triggers the release of pro-inflammatory cytokines and activation of adaptive immune response to eliminate invading pathogens [81]. TLR4 detects danger signals which are products of inflammation and tissue injury. TLR4 is highly expressed by immune cells in the CNS and is involved in MS neuropathology [82]. Importantly, TLR4 agonists participate in the amplification of harmful inflammatory responses. Fibrinogen activates the immune response by stimulating TLR4 which is involved in immune activation [79]. Fibrinogen triggers the release of chemokines like macrophage inflammatory protein 1 alpha (MIP-1α) from macrophages by activating TLR4 [83]. In addition, the fibrinogen-TLR4 complex promotes the release of pro-inflammatory cytokines leading to the propagation of inflammatory disorders [84]. Fibrinogen can act synergistically with MIP-1β in the propagation of inflammation in patients with acute coronary syndrome [85]. Notably, MIP-1α is augmented in MS lesions and correlated with disease severity [86]. Therefore, fibrinogen is regarded as a component of damage-associated molecular patterns involved in the early immune response [87]. The findings of these studies suggest that the fibrinogen-TLR4 complex plays a critical role in the pathogenesis of MS.

#### NF-κB

NF-κB is a DNA-binding protein necessary for the transcription of chemokines and pro-inflammatory cytokines. Predominantly, immune deregulation and increased expression of NF-κB are linked with the progression of neuronal injury, neuroinflammation, and the development of neurodegeneration [88]. NF-κB is overstated in MS leading to immune dysregulation and induction of the release of pro-inflammatory cytokines. Inhibition of the NF-κB signaling pathway by dimethyl fumarate may reduce MS severity [89]. It has been observed that native and memory B cells from MS patients have a higher level of phosphorylated NF-κB which was inhibited by mycophenolate [90]. In addition, glatiramer attenuates the activation of NF-κB by inhibiting CD40 which is over-activated in MS [90].

Fibrinogen plays an integral role in the regulation of immune response and release of pro-inflammatory cytokines through NF-κB activation [91]. In vitro study demonstrated that fibrinogen promotes the release of IL-8 and monocyte chemoattractant protein 1 (MCP-1) in a concentration-dependent manner by activating the NF-κB signaling pathway [91]. Inhibition of IκB kinase by herbal parthenolide prevents expression and the release of MCP-1 under the effect of fibrinogen. Guo et al. [91] suggest a role of NF-κB in mediating the inflammatory effect of fibrinogen. Rubel et al. [92] revealed that fibrinogen through interaction with CD11b/CD18 can activate the apoptotic pathway in human neutrophils. Furthermore, fibrinogen triggers the development of endothelial dysfunction by increasing the expression of adhesion molecules like VCAM-1 and intercellular adhesion molecule 1 (ICAM-1) which promote the expression of NF-κB [93]. These verdicts indicated that fibrinogen triggers NF-κB activation, and accordingly may increase inflammatory reactions in MS.

#### NLRP3 Inflammasome

One of the most important inflammatory signaling pathways is the NLRP3 inflammasome which is concerned in the activation of caspase-1, and maturation of IL-1β and IL-18 [94]. NLRP3 inflammasome is triggered by miscellaneous stimuli and inflammatory signaling pathways like NF-κB [94]. NLRP3 inflammasome is intricate in the pathogenesis of neuroinflammation, and the development of neurodegeneration [95]. NLRP3 inflammasome is also exaggerated and linked with the severity of MS [96]. NLRP3 inflammasome within activated microglia promotes the expression and the release of IL-1β and IL-18. Confirmation from preclinical and clinical findings illustrated that aberrant activation of NLRP3 inflammasome is associated with the pathogenesis of MS. Over-activation of NLRP3 inflammasome in MS is apparent by increasing IL-1β CSF levels in severely affected patients [97]. Targeting of NLRP3 inflammasome by specific inhibitors can reduce MS severity [97].

It has been shown that fibrinogen and fibrinogen-like protein 2 through activation of TLR4 promotes the expression of NLRP3 inflammasome and the release of pro-inflammatory cytokines [98]. Roseborough et al. [99] showed that fibrinogen propagates pro-inflammatory signaling by priming microglial NLRP3 inflammasome in a dose-dependent manner. In addition, extracellular vesicles released activated microglia by fibrinogen priming signaling to naïve cells. These extracellular vesicles from injured microglia are increased in the peripheral circulation and correlated with increasing IL-6 and IL-1β which are a biomarker of NLRP3 inflammasome activity [99]. Therefore, a higher level of fibrinogen level in MS induces more inflammatory changes and the development of neuroinflammation.

### Neuroinflammation

It has been reported by different studies that neuroinflammation is connected with the progression of diverse neurodegenerative disorders [96, 100]. Lymphocytes in the CNS activate inflammatory disorders and the progress of neuroinflammation [94]. Neuroinflammation in the early stage of MS can cause synaptopathy independent of the demyelination process, and this may explain cognitive dysfunction in the early phase of MS patients. However, in the late phase of MS, overstatement of immune disturbance and progress neuroinflammation stimulate MS pathogenesis [101]. Notably, cholinergic activity is impaired in MS patients that control the activity and response of immune cells. A decrease in the acetylcholine level in the immune cells promotes the release of pro-inflammatory cytokines with the progress of neuroinflammation [102, 103]. Of interest, fibrinogen is regarded as a potent inducer of neuroinflammation in different neurodegenerative disorders including AD, MS, and traumatic brain injury [104]. Hyperfibrinogenemia and fibrin deposits are associated with memory deficit and cognitive dysfunction in AD. Fibrinogen activates astrocytes, microglia, and neurons leading to neuroinflammation and neuronal injury [104]. Fibrinogen binds CD11b/CD18 which is also called Mac-1 on monocytes, macrophages, and microglia leading to the release of ROS in experimental autoimmune encephalomyelitis [105]. In addition, fibrinogen triggers neuroinflammation through the activation of platelets [106]. Remarkably, fibrinogen enhances the recruitment of peripheral monocytes and interaction with myelin-specific antigens [107]. Therefore, genetic deletion of the fibrinogen gene reduces neuroinflammation and demyelination in transgenic mice with MS [108]. Likewise, genetic deletion or use of specific inhibitors against CD11b/CD18 attenuates the development of experimental autoimmune encephalomyelitis in mice [109]. According to these findings, inhibition of fibrinogen could be a novel therapeutic strategy against neuroinflammation in MS.

### Brain-Derived Neurotrophic Factor

Brain-derived neurotrophic factor (BDNF) also called abrineurin is a protein encoded by the BDNF gene [110]. BDNF is a member of neurotrophin growth factors found in the brain and periphery. BDNF acts on tropomyosin receptor kinase B (TrkB) receptors which are catalytic receptors for different neurotrophins involved in the growth and differentiation of cells [111]. Neuronal TrkB is highly active in the hippocampus, basal forebrain, and cerebral cortex. In addition, BDNF also activates low-affinity nerve growth factor receptor (LNGFR) whose function is not fully elucidated [112].

BDNF levels may decrease in MS due to the advanced neurodegeneration process [113]. A case-control study on 22 MS patients compared to 19 healthy controls exposed that BDNF serum level was decreased in MS patients compared to the controls [113]. Though, a recent study detected that BDNF serum level was not considerably reduced in MS patients compared to healthy controls [114]. In addition, a systemic review comprising 30 studies (689 MS patients and 583 healthy controls) discovered that BDNF serum level was decreased in MS patients compared to healthy controls [115].

**Fig. 5:**
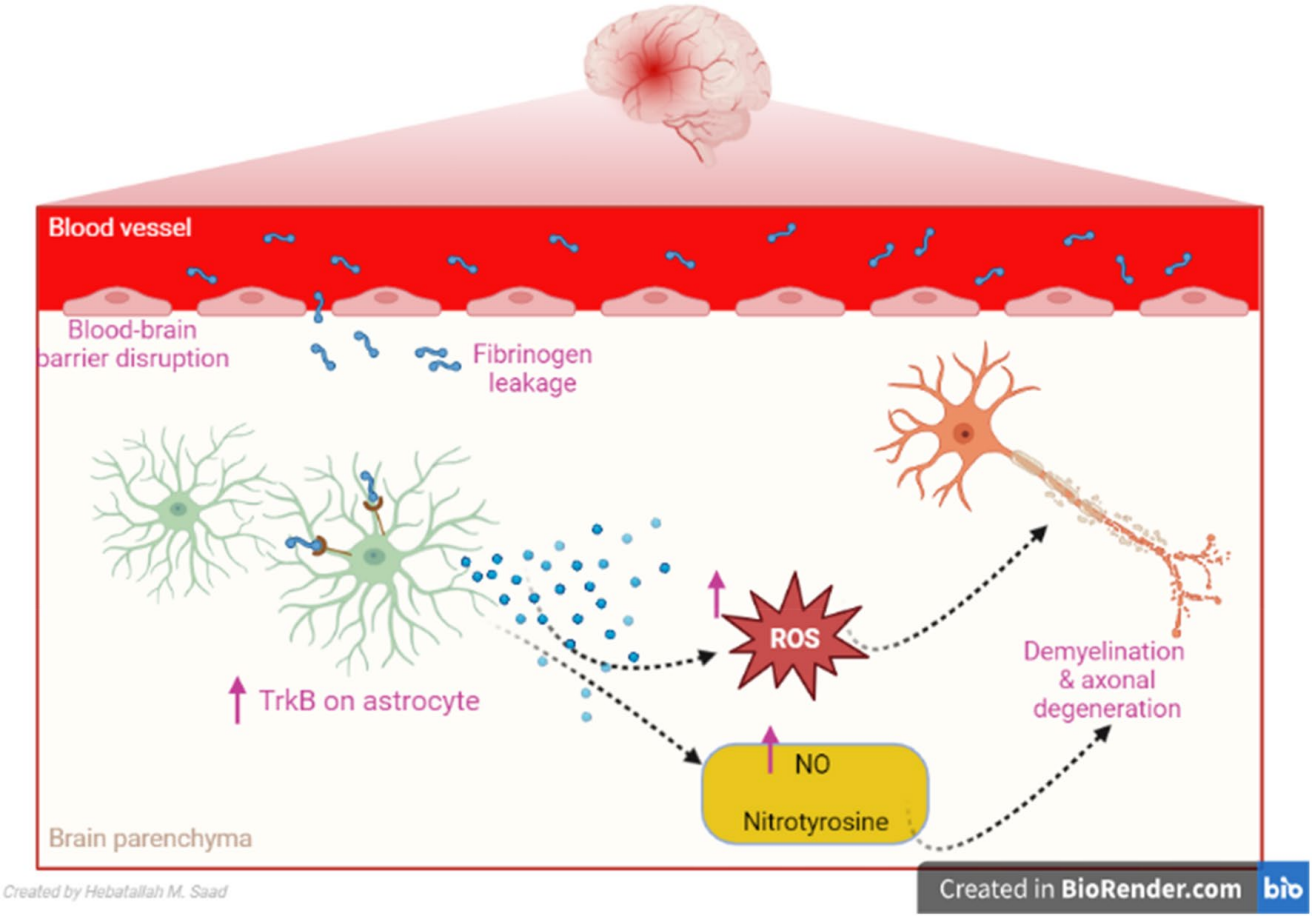
Tropomyosin receptor kinase B (TrkB) in multiple sclerosis (MS): Fibrinogen increases the expression of tropomyosin receptor kinase B (TrkB) receptors in astrocytes. Over-expression of astrocytes TrkB is linked with morphological changes of astrocytes with subsequent over-production of nitric oxide (NO), the release of nitrotyrosine and reactive oxygen species (ROS) which promote MS

It has been shown that fibrinogen increases the expression of TrkB in astrocytes in a dose-dependent manner [104]. However, over-expression of astrocytes TrkB is linked with morphological changes of astrocytes [116] with subsequent over-production of nitric oxide (NO), the release of nitrotyrosine and ROS which promote neurodegeneration [117]. These findings proposed that overexpression of TrkB by fibrinogen induces neurodegeneration in MS (Fig. 5).

## Targeting of Fibrinogen in MS

It has been shown platelet activity is increased in MS patients due to the over-expression of platelet GPIIb/IIIa receptors which are activated by fibrinogen. Platelet over-activity in MS promotes the formation of platelet microparticles and platelet aggregate which increase cardiovascular complications [118]. Platelet abnormality is developed in MS that causes more inflammation in the neurovascular unit. Platelets are the main source of IL-1α which affect brain endothelium and enhance entry of immune cells into the CNS causing cerebrovascular inflammation [119]. In addition, platelets contribute to the progression of inflammation in the early stage of MS [120]. Therefore, GPIIb/IIIa receptor antagonists may reduce thrombotic events in MS patients.

Of note, fibrinogen binds CD11b/CD18 also called complement receptor type 3 (CR3) which is also called Mac-1 on monocytes, macrophages and microglia to induce the release of ROS in MS [105]. Thus, inhibition of CD11b/CD18 receptors by specific antagonists may attenuate fibrinogen-induced neuroinflammation and demyelination. However, most CD11b/CD18 receptors were used in experimental studies but are not confirmed clinically. Therefore, the repurposing of clinical approval drugs which affect the expression of CD11b/CD18 is promising.

### Metformin

Metformin is an insulin-sensitizing drug that improves peripheral insulin sensitivity and reduces hepatic glucose uptake. It is used as a first line in the management of type 2 diabetes mellitus (T2DM) [121, 122]. In addition, metformin has a neuroprotective effect against the development and progression of AD [123]. As well, metformin reduces neuroinflammation and demyelination in MS [124]. Different preclinical and clinical studies (Table 1) indicated that metformin is effective in the management of MS. Metformin attenuates the induction of experimental autoimmune encephalomyelitis by restricting the infiltration of mononuclear cells into the CNS, down-regulating the expression of pro-inflammatory cytokines [gamma interferone (IFN-γ), tumor necrosis factor alpha (TNF-α), IL-6, IL-17, and inducible NO synthase (iNOS)], cell adhesion molecules, matrix metalloproteinase 9, and chemokine [125]. Metformin inhibited T cell-mediated immune responses including Ag-specific recall responses and production of Th1 or Th17 cytokines, while it induced the generation of IL-10 in spleen cells of treated experimental autoimmune encephalomyelitis animals [125]. Metformin reduced Th17 and increased Treg cell percentages along with the levels of associated cytokines. Molecules involved in cellular metabolism were altered in mice with experimental autoimmune encephalomyelitis. Suppressed activation of the mechanistic target of rapamycin (mTOR) and its downstream target, hypoxia-inducible factor 1 α (HIF-1α), likely mediated the protective effects of metformin [126]. Treatment with metformin has beneficial anti-inflammatory effects in patients with MS by a significant increase in the number and regulatory functions of CD4+ and CD25+, and regulatory T cells compared with controls [127].

**Table 1:** Role of metformin in multiple sclerosis (MS)

Study type	Findings	References
An experimental study	Metformin 100 mg/kg/day attenuates experimental autoimmune encephalomyelitis in mice by down-regulating the expression of pro-inflammatory cytokines, inducible nitric oxide synthase (iNOS)), cell adhesion molecules, and chemokine	Nath et al. [125]
An experimental study	Metformin 100 mg/kg/day in mice reduced Th17 and increased Treg cell percentages along with the levels of associated cytokines in experimental autoimmune encephalomyelitis	Sun et al. [126]
A case control study	Metformin 850–1500 mg/day has anti-inflammatory effects in patients with MS by increasig in the number and regulatory functions of regulatory T cells compared with controls	Negrotto et al. [127]
A placebo-controlled clinical trial	Metformin 850 mg/twice daily improves fibrinolysis in obese patients	Charles et al. [144]
An experimental study	Metformin therapy 100 mg/kg/day significantly alleviated reactive microgliosis and astrogliosis in mice with experimental autoimmune encephalomyelitis	Abdi et al. [145]

A previous comparative study illustrated that metformin therapy reduced fibrinogen levels in obese patients with T2DM [128]. Baptista and colleagues observed that metformin therapy reduced fibrinogen levels and inflammatory biomarkers in olanzapine-induced metabolic dysfunction in patients with schizophrenia [129]. However, a systematic review involving 9 randomized, placebo-controlled trials of 2302 patients showed that metformin therapy did not reduce fibrinogen levels significantly [130]. Though, this systematic review did not involve clinical studies regarding fibrinogen levels and the effects of metformin in T2DM patients. In progressive MS, fibrinogen level is progressively increased in the cerebral cortex and CSF in patients with MS [131]. Fibrinogen not only induces demyelination but also inhibits remyelination and neurogenesis by inducing the expression of bone morphogenic protein (BMP) receptors which inhibit remyelination [132]. Therefore, inhibiting the expression of BMP may prevent the inhibitory effect of fibrinogen on the remyelination process. It has been shown that metformin inhibits the expression of BMP and its signaling in trauma-induced ossification [133]. In addition, metformin through an AMP-activated protein kinase (AMPK)-dependent pathway negatively regulates BMP signaling [134]. Therefore, metformin through inhibition of fibrinogen and related BMP signaling may attenuate the development and progression of MS.

### Statins

Statins are a class of lipid-lowering agents used in the management of hypercholesterolemia in patients with cardiovascular complications [134]. Statins act by inhibiting hyroxymethylglutaryl-CoA (HMG-CoA) a rate-limiting enzyme in denovo cholesterol biosynthesis [135]. Of note, statins have neuroprotective effects against different neurodegenerative diseases [1]. In addition, different studies revealed that statins are effective against MS neuropathology (Table 2) [136, 137]. However, there is a conflict regarding the beneficial effects of statins in MS [138]. Statins are safe drugs, widely used in patients with cardiovascular complications, and have pleiotropic effects. Therefore, statins may affect fibrinogen which is implicated in MS neuropathology. It has been reported that atorvastatin reduced fibrinogen levels in patients with hyperlipidemia [139]. However, fluvastatin increases fibrinogen levels [140]. Therefore, there is a controversy regarding the effect of statins on fibrinogen level. Recently, a cohort study illustrated that rosuvastatin fibrinogen level had significant fibrinolytic effects [141].

**Table 2:** Role of statins in multiple sclerosis (MS)

Study type	Findings	References
A placebo-controlled clinical Trial	Different statins for 24 months reduced disease severity in patients with MS	Wang et al. [137]
A review	Statins may have neuroprotective and neuro-repairing effects in clinical MS and experimental autoimmune encephalomyelitis	Xu et al. [146]
Double-blind randomized controlled trial	Atorvastatin 40 mg/kg for 18 months reduced severity and relapse in MS patients	Ghasami et al. [147]
Multicenter double-blind placebo-controlled phase II Trials	Simvastatin 80 mg/kg for 24 months reduced severity and relapse in MS patients	Chataway et al. [148]
A systematic review and meta-analysis	No benefit from statin treatment as an add-on to interferon beta (IFN-β) in MS patients	Stefanou et al. [149]

Despite these controversies, statins reduce the expression of CD11b/CD18 thereby reducing monocyte activation [142]. In addition, statins can mitigate neuroinflammation in different neurological disorders by inhibiting the expression of CD11b/CD18 [143]. Therefore, statins seem to not affect fibrinogen levels but decrease its effect on the brain by reducing CD11b/CD18.

Taken together, both statins and metformin which modulate the fibrinogen pathway and inflammatory reactions in MS could be adjuvant treatments with immunomodulatory agents in the management of MS. In this bargain, preclinical and large-scale prospective studies are recommended in this regard.

## Conclusions

MS is an autoimmune demyelinating neurodegenerative disease of the CNS due to injury of myelin sheath by immune cells and inhibition of the production of myelin. The clotting factor fibrinogen is intricate in the pathogenesis of MS by triggering microglia and the progress of neuroinflammation. Fibrinogen level is correlated with MS severity; therefore, inhibition of the fibrinogen cascade may reduce MS neuropathology.

Deposition of fibrinogen in the CNS precedes neuroinflammation in MS. Accumulation of fibrinogen in the CNS may occur due to disruption of BBB integrity in MS. Disturbance of BBB integrity in MS may precede the development of brain lesions that may promote the entrance of plasma proteins including fibrinogen. Fibrinogen acts as transduction and increases microglia activation via triggering expression of CD11b/CD18. Likewise, brain fibrinogen induces the progression of inflammation, oxidative stress, and neuronal injury. Interestingly, brain fibrinogen impairs the remyelination process by inhibiting the differentiation of oligodendrocyte precursor cells. Moreover, increased fibrinogen level in the proteome of platelets is correlated with the progression of MS. These findings proposed that fibrinogen is associated with MS neuropathology through interruption of BBB integrity, induction of neuroinflammation, and demyelination with inhibition of the remyelination process by suppressing oligodendrocytes. Therefore, targeting of fibrinogen and/or CD11b/CD18 receptors by metformin and statins may reduce MS neuropathology.

Taken together, inhibition expression of CD11b/CD18 receptors by metformin and statins decreases the pro-inflammatory effect of fibrinogen on microglia which is involved in the progression of MS.

## Data Availability

All data supporting the findings of this study are available in the paper.
